# 4-{[(*E*)-2,3-Dihy­droxy­benzyl­idene]amino}-*N*-(5-methyl-1,2-oxazol-3-yl)benzene­sulfonamide

**DOI:** 10.1107/S1600536812026657

**Published:** 2012-06-16

**Authors:** M. Nawaz Tahir, Abdul Haleem Khan, Mohammad S. Iqbal, Christy Munir, Tariq Aziz

**Affiliations:** a Department of Physics, University of Sargodha, Sargodha, Pakistan; b Department of Pharmacy Services, Jinnah Hospital, Lahore, Pakistan; c Department of Chemistry, Forman Christian College, Lahore 54600, Pakistan

## Abstract

In the title compound, C_17_H_15_N_3_O_5_S, the 2,3-dihy­droxy­benzaldehyde unit is oriented at a dihedral angles of 16.83 (10) and 78.87 (6)° with the anilinic and 5-methyl-1,2-oxazol-3-amine groups, respectively. An *S*(6) loop exists due to intramolecular O—H⋯N hydrogen bonding. In the crystal, inversion dimers with *R*_2_^2^(8) rings are formed due to N—H⋯N hydrogen bonding between the 5-methyl-1,2-oxazol-3-amine groups. These dimers are inter­linked by O—H⋯O hydrogen bonds, forming chains along [101] and resulting in *R*_2_^2^(26) rings. π–π inter­actions occur between the central benzene rings with a centroid–centroid distance of 3.7928 (16) Å.

## Related literature

For related structures, see: Ebenezer & Muthiah (2010 ▶); Yildiz *et al.* (2010 ▶). For graph-set notation, see: Bernstein *et al.* (1995 ▶).
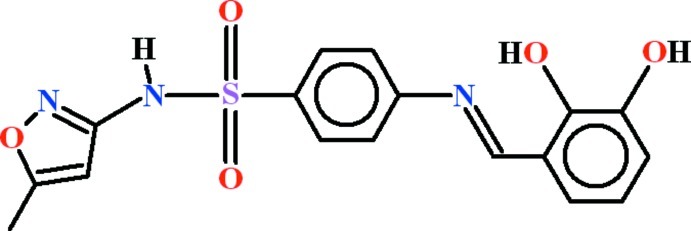


## Experimental

### 

#### Crystal data

C_17_H_15_N_3_O_5_S
*M* *_r_* = 373.38
Triclinic, 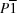

*a* = 7.1881 (6) Å
*b* = 10.6682 (10) Å
*c* = 11.6865 (9) Å
α = 92.181 (4)°
β = 99.776 (4)°
γ = 99.606 (5)°
*V* = 868.74 (13) Å^3^
*Z* = 2
Mo *K*α radiation
μ = 0.22 mm^−1^
*T* = 296 K
0.25 × 0.18 × 0.16 mm

#### Data collection

Bruker Kappa APEXII CCD diffractometer
Absorption correction: multi-scan (*SADABS*; Bruker, 2005 ▶) *T*_min_ = 0.957, *T*_max_ = 0.966
12511 measured reflections
3389 independent reflections
2004 reflections with *I* > 2σ(*I*)
*R*_int_ = 0.048

#### Refinement

*R*[*F* ^2^ > 2σ(*F* ^2^)] = 0.049
*wR*(*F* ^2^) = 0.121
*S* = 1.00
3389 reflections
245 parameters
All H-atom parameters refined
Δρ_max_ = 0.19 e Å^−3^
Δρ_min_ = −0.27 e Å^−3^

### 

Data collection: *APEX2* (Bruker, 2007 ▶); cell refinement: *SAINT* (Bruker, 2007 ▶); data reduction: *SAINT*; program(s) used to solve structure: *SHELXS97* (Sheldrick, 2008 ▶); program(s) used to refine structure: *SHELXL97* (Sheldrick, 2008 ▶); molecular graphics: *ORTEP-3 for Windows* (Farrugia, 1997 ▶) and *PLATON* (Spek, 2009 ▶); software used to prepare material for publication: *WinGX* (Farrugia, 1999 ▶) and *PLATON*.

## Supplementary Material

Crystal structure: contains datablock(s) global, I. DOI: 10.1107/S1600536812026657/bq2368sup1.cif

Structure factors: contains datablock(s) I. DOI: 10.1107/S1600536812026657/bq2368Isup2.hkl

Supplementary material file. DOI: 10.1107/S1600536812026657/bq2368Isup3.cml

Additional supplementary materials:  crystallographic information; 3D view; checkCIF report

## Figures and Tables

**Table 1:** Hydrogen-bond geometry (Å, °)

*D*—H⋯*A*	*D*—H	H⋯*A*	*D*⋯*A*	*D*—H⋯*A*
O1—H1⋯N1	0.99 (4)	1.66 (4)	2.588 (3)	155 (3)
O2—H2⋯O4^i^	0.79 (4)	2.11 (5)	2.848 (3)	154 (5)
N2—H2*A*⋯N3^ii^	0.84 (3)	2.05 (3)	2.881 (3)	172 (3)

Symmetry codes: (i) 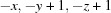; (ii) 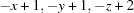.

**Fig. 1.**
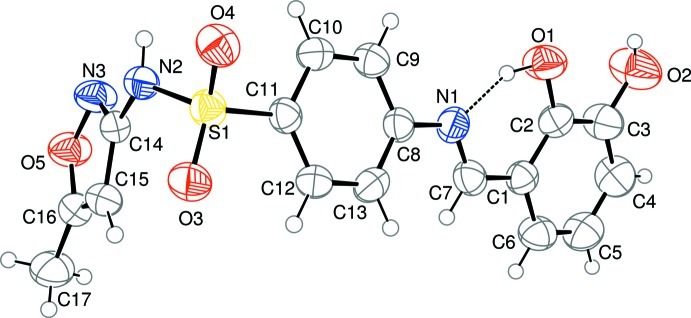
View of the title compound with the atom numbering scheme. The thermal ellipsoids are drawn at the 50% probability level. The dotted line represent the intramolecular H-bond.

**Fig. 2.**
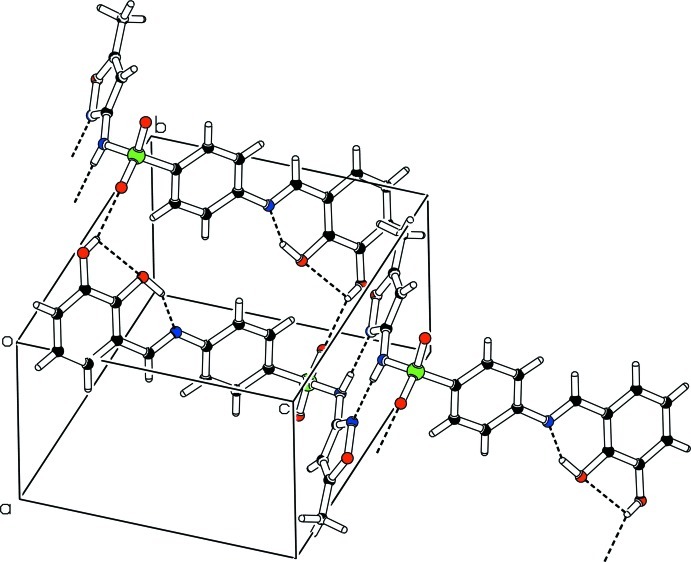
The partial packing (PLATON; Spek, 2009) which shows that molecules form dimers.

## supplementary crystallographic information

### Comment

The title compound (I), (Fig. 1) has been synthesized for the biological studies and forming different metal complexes. The crystal structures of 4-((2-hydroxybenzylidene)amino)-*N*-(5-methyl-1, 2-oxazol-3-yl)benzenesulfonamide (Ebenezer & Muthiah, 2010) and 4-((2-hydroxy-3-methoxybenzylidene)amino)-*N*-(5-methyl-1,2-oxazol-3-yl) benzenesulfonamide (Yildiz *et al.*, 2010) have been published previously which are related to the title compound.

In (I), the parts of 2,3-dihydroxybenzaldehyde A (C1—C7/O1/O2), annilinic group B (C8—C13/N1) and 5-methyl-1,2-oxazol-3-amine C (C14—C17/N2/N3/O5) are planar with r. m. s. deviations of 0.0057 Å, 0.0055 Å and 0.0198 Å, respectively. The dihedral angles between A/B, A/C and B/C are 16.83 (10)°, 78.87 (6)° and 80.41 (6)°, respectively. The sulfonyl group D (O3/S1/O4) is of course planar. The dihedral angles between A/D, B/D and C/D are 49.52 (8)°, 52.65 (9)° and 29.35 (14)°, respectively. In (I), *S*(6) ring motif (Bernstein *et al.*, 1995) is present due to H-bonding of O—H···N type, (Table 1, Fig. 1). The molecules are dimerized from 5-methyl-1,2-oxazol-3-amine groups due to H-bondings of N—H···N type with *R*_2_^2^(8) ring motif (Table 1, Fig. 2). The dimers are interlinked due to H-bondings of O—H···O type with *R*_2_^2^(26) ring motif (Table 1, Fig. 2) and therefore, one-dimensional polymeric chains is formed along the base vector [1 0 1]. There exist π–π interaction between *Cg*1···*Cg*1^i^ [i = 1 - *x*, 1 - *y*, 1 - *z*] at a distance of 3.7928 (16) Å, where *Cg*1 is the centroid of benzene ring (C8—C13).

### Experimental

Equimolar quantities of 4-amino-*N*-(5-methylisoxazol-3-yl)-benzenesulfonamide (Sulfamethoxazole) and 2,3-dihydroxybenzaldehyde were refluxed in methanol along with few drops of acetic acid as catalyst for 1 h. The solution was kept at room temperature which afforded red prisms after two days.

### Refinement

In the absence of anomalous scattering factor, the Friedel pairs were merged. The coordinates of amide and hydroxy H-atoms were refined. The H-atoms were positioned geometrically (C–H = 0.93–0.96 Å) and refined as riding with *U*_iso_(H) = *xU*_eq_(C, N, O), where *x* = 1.5 for hydroxy & methyl and *x* = 1.2 for other H-atoms.

### Crystal data

**Table:**

C_17_H_15_N_3_O_5_S	*Z* = 2
*M**_r_* = 373.38	*F*(000) = 388
Triclinic, *P*1	*D*_x_ = 1.427 Mg m^−^^3^
Hall symbol: -P 1	Mo *K*α radiation, λ = 0.71073 Å
*a* = 7.1881 (6) Å	Cell parameters from 2704 reflections
*b* = 10.6682 (10) Å	θ = 1.8–26.0°
*c* = 11.6865 (9) Å	µ = 0.22 mm^−^^1^
α = 92.181 (4)°	*T* = 296 K
β = 99.776 (4)°	Prism, red
γ = 99.606 (5)°	0.25 × 0.18 × 0.16 mm
*V* = 868.74 (13) Å^3^	

### Data collection

**Table:**

Bruker Kappa APEXII CCD diffractometer	3389 independent reflections
Radiation source: fine-focus sealed tube	2004 reflections with *I* > 2σ(*I*)
Graphite monochromator	*R*_int_ = 0.048
Detector resolution: 8.00 pixels mm^-1^	θ_max_ = 26.0°, θ_min_ = 1.9°
ω scans	*h* = −8→8
Absorption correction: multi-scan (*SADABS*; Bruker, 2005)	*k* = −13→13
*T*_min_ = 0.957, *T*_max_ = 0.966	*l* = −14→14
12511 measured reflections	

### Refinement

**Table:**

Refinement on *F*^2^	Primary atom site location: structure-invariant direct methods
Least-squares matrix: full	Secondary atom site location: difference Fourier map
*R*[*F*^2^ > 2σ(*F*^2^)] = 0.049	Hydrogen site location: inferred from neighbouring sites
*wR*(*F*^2^) = 0.121	All H-atom parameters refined
*S* = 1.00	*w* = 1/[σ^2^(*F*_o_^2^) + (0.0529*P*)^2^] where *P* = (*F*_o_^2^ + 2*F*_c_^2^)/3
3389 reflections	(Δ/σ)_max_ < 0.001
245 parameters	Δρ_max_ = 0.19 e Å^−^^3^
0 restraints	Δρ_min_ = −0.27 e Å^−^^3^

### Special details

**Table:**

Geometry. Bond distances, angles *etc*. have been calculated using the rounded fractional coordinates. All su's are estimated from the variances of the (full) variance-covariance matrix. The cell e.s.d.'s are taken into account in the estimation of distances, angles and torsion angles
Refinement. Refinement of *F*^2^ against ALL reflections. The weighted *R*-factor *wR* and goodness of fit *S* are based on *F*^2^, conventional *R*-factors *R* are based on *F*, with *F* set to zero for negative *F*^2^. The threshold expression of *F*^2^ > σ(*F*^2^) is used only for calculating *R*-factors(gt) *etc*. and is not relevant to the choice of reflections for refinement. *R*-factors based on *F*^2^ are statistically about twice as large as those based on *F*, and *R*- factors based on ALL data will be even larger.

### Fractional atomic coordinates and isotropic or equivalent isotropic displacement parameters (Å^2^)

**Table:**

	*x*	*y*	*z*	*U*_iso_*/*U*_eq_	
S1	0.72001 (10)	0.57361 (7)	0.76687 (5)	0.0470 (3)	
O1	−0.2827 (3)	0.2045 (2)	0.35929 (18)	0.0731 (8)	
O2	−0.5837 (4)	0.0532 (2)	0.2208 (3)	0.1008 (11)	
O3	0.8978 (2)	0.54264 (18)	0.74645 (14)	0.0586 (7)	
O4	0.6855 (3)	0.70107 (17)	0.75951 (15)	0.0566 (7)	
O5	0.6964 (3)	0.27141 (18)	1.05698 (15)	0.0627 (8)	
N1	0.0732 (3)	0.2368 (2)	0.45973 (17)	0.0504 (8)	
N2	0.6991 (3)	0.5411 (2)	0.89993 (17)	0.0455 (8)	
N3	0.6258 (3)	0.3814 (2)	1.02145 (19)	0.0578 (9)	
C1	−0.0786 (4)	0.0495 (3)	0.3390 (2)	0.0482 (10)	
C2	−0.2552 (4)	0.0909 (3)	0.3158 (2)	0.0539 (11)	
C3	−0.4110 (4)	0.0144 (3)	0.2441 (3)	0.0661 (12)	
C4	−0.3908 (5)	−0.1007 (3)	0.1984 (3)	0.0723 (12)	
C5	−0.2166 (5)	−0.1433 (3)	0.2209 (3)	0.0711 (14)	
C6	−0.0622 (5)	−0.0683 (3)	0.2899 (2)	0.0616 (11)	
C7	0.0824 (4)	0.1275 (3)	0.4135 (2)	0.0534 (11)	
C8	0.2336 (4)	0.3133 (3)	0.5313 (2)	0.0454 (9)	
C9	0.1979 (4)	0.4123 (3)	0.6001 (2)	0.0505 (10)	
C10	0.3444 (4)	0.4908 (3)	0.6723 (2)	0.0498 (10)	
C11	0.5320 (3)	0.4718 (2)	0.67560 (19)	0.0433 (9)	
C12	0.5690 (4)	0.3743 (3)	0.6069 (2)	0.0536 (10)	
C13	0.4211 (4)	0.2965 (3)	0.5342 (2)	0.0561 (11)	
C14	0.7297 (3)	0.4260 (2)	0.9455 (2)	0.0409 (9)	
C15	0.8621 (4)	0.3492 (3)	0.9277 (2)	0.0497 (10)	
C16	0.8378 (4)	0.2557 (3)	0.9987 (2)	0.0507 (10)	
C17	0.9254 (5)	0.1416 (3)	1.0241 (3)	0.0722 (14)	
H1	−0.157 (5)	0.237 (3)	0.409 (3)	0.1096*	
H2	−0.575 (7)	0.128 (4)	0.232 (4)	0.1510*	
H2A	0.610 (4)	0.571 (3)	0.923 (2)	0.0545*	
H4	−0.49539	−0.15176	0.15139	0.0867*	
H5	−0.20499	−0.22224	0.18912	0.0852*	
H6	0.05487	−0.09628	0.30425	0.0738*	
H7	0.19768	0.09741	0.42876	0.0641*	
H9	0.07257	0.42547	0.59707	0.0606*	
H10	0.31878	0.55625	0.71871	0.0597*	
H12	0.69437	0.36111	0.60986	0.0643*	
H13	0.44706	0.23211	0.48657	0.0669*	
H15	0.94902	0.36074	0.87682	0.0596*	
H17A	0.95686	0.13684	1.10685	0.1083*	
H17B	1.03998	0.14728	0.99126	0.1083*	
H17C	0.83640	0.06668	0.99078	0.1083*	

### Atomic displacement parameters (Å^2^)

**Table:**

	*U*^11^	*U*^22^	*U*^33^	*U*^12^	*U*^13^	*U*^23^
S1	0.0425 (4)	0.0536 (5)	0.0455 (4)	0.0087 (3)	0.0085 (3)	0.0062 (3)
O1	0.0544 (14)	0.0700 (15)	0.0902 (15)	0.0144 (12)	0.0028 (11)	−0.0234 (12)
O2	0.0566 (16)	0.0831 (19)	0.148 (2)	0.0095 (15)	−0.0128 (14)	−0.0255 (18)
O3	0.0377 (11)	0.0792 (15)	0.0621 (11)	0.0119 (10)	0.0168 (8)	0.0039 (10)
O4	0.0608 (13)	0.0453 (12)	0.0637 (12)	0.0103 (10)	0.0087 (9)	0.0122 (9)
O5	0.0777 (15)	0.0611 (14)	0.0611 (12)	0.0310 (12)	0.0245 (10)	0.0172 (10)
N1	0.0495 (15)	0.0590 (16)	0.0416 (12)	0.0078 (12)	0.0073 (10)	0.0002 (12)
N2	0.0463 (15)	0.0529 (15)	0.0410 (12)	0.0177 (12)	0.0094 (10)	0.0050 (11)
N3	0.0693 (17)	0.0581 (16)	0.0585 (14)	0.0326 (14)	0.0229 (12)	0.0175 (12)
C1	0.0533 (18)	0.0474 (18)	0.0447 (15)	0.0087 (15)	0.0107 (12)	0.0060 (13)
C2	0.058 (2)	0.0494 (19)	0.0536 (16)	0.0082 (16)	0.0106 (13)	−0.0029 (14)
C3	0.054 (2)	0.057 (2)	0.083 (2)	0.0067 (17)	0.0054 (16)	−0.0046 (18)
C4	0.072 (2)	0.051 (2)	0.084 (2)	−0.0021 (18)	0.0013 (18)	−0.0057 (18)
C5	0.090 (3)	0.047 (2)	0.076 (2)	0.010 (2)	0.0170 (19)	−0.0004 (17)
C6	0.071 (2)	0.0500 (19)	0.0683 (19)	0.0191 (17)	0.0161 (16)	0.0089 (16)
C7	0.0518 (19)	0.063 (2)	0.0486 (16)	0.0157 (16)	0.0105 (13)	0.0131 (15)
C8	0.0436 (17)	0.0556 (18)	0.0382 (14)	0.0119 (14)	0.0065 (12)	0.0068 (13)
C9	0.0434 (17)	0.0560 (19)	0.0530 (16)	0.0113 (15)	0.0089 (13)	0.0041 (14)
C10	0.0477 (18)	0.0520 (18)	0.0518 (16)	0.0141 (14)	0.0109 (13)	−0.0016 (14)
C11	0.0425 (16)	0.0496 (17)	0.0388 (13)	0.0097 (13)	0.0072 (11)	0.0083 (12)
C12	0.0431 (17)	0.072 (2)	0.0496 (16)	0.0197 (16)	0.0110 (13)	−0.0007 (15)
C13	0.056 (2)	0.068 (2)	0.0453 (15)	0.0146 (16)	0.0111 (13)	−0.0086 (14)
C14	0.0392 (15)	0.0433 (16)	0.0387 (14)	0.0112 (13)	0.0002 (11)	−0.0024 (12)
C15	0.0443 (17)	0.0561 (19)	0.0518 (16)	0.0171 (15)	0.0094 (12)	0.0038 (14)
C16	0.0521 (18)	0.0532 (19)	0.0472 (15)	0.0178 (15)	0.0025 (13)	−0.0011 (14)
C17	0.091 (3)	0.064 (2)	0.072 (2)	0.040 (2)	0.0165 (17)	0.0122 (17)

### Geometric parameters (Å, º)

**Table:**

S1—O3	1.4275 (17)	C8—C9	1.387 (4)
S1—O4	1.426 (2)	C8—C13	1.384 (4)
S1—N2	1.632 (2)	C9—C10	1.368 (4)
S1—C11	1.754 (2)	C10—C11	1.391 (4)
O1—C2	1.354 (4)	C11—C12	1.378 (4)
O2—C3	1.361 (4)	C12—C13	1.373 (4)
O5—N3	1.404 (3)	C14—C15	1.391 (4)
O5—C16	1.345 (4)	C15—C16	1.330 (4)
O1—H1	0.99 (4)	C16—C17	1.479 (5)
O2—H2	0.79 (4)	C4—H4	0.9300
N1—C8	1.415 (4)	C5—H5	0.9300
N1—C7	1.283 (4)	C6—H6	0.9300
N2—C14	1.393 (3)	C7—H7	0.9300
N3—C14	1.308 (3)	C9—H9	0.9300
N2—H2A	0.84 (3)	C10—H10	0.9300
C1—C2	1.400 (4)	C12—H12	0.9300
C1—C7	1.439 (4)	C13—H13	0.9300
C1—C6	1.393 (4)	C15—H15	0.9300
C2—C3	1.395 (4)	C17—H17A	0.9600
C3—C4	1.360 (5)	C17—H17B	0.9600
C4—C5	1.390 (5)	C17—H17C	0.9600
C5—C6	1.369 (5)		
			
O3—S1—O4	120.39 (13)	S1—C11—C10	119.41 (18)
O3—S1—N2	107.74 (11)	C11—C12—C13	120.1 (3)
O3—S1—C11	108.71 (11)	C8—C13—C12	120.4 (3)
O4—S1—N2	104.10 (11)	N2—C14—N3	117.6 (2)
O4—S1—C11	108.69 (11)	N2—C14—C15	130.5 (2)
N2—S1—C11	106.32 (11)	N3—C14—C15	111.9 (2)
N3—O5—C16	108.6 (2)	C14—C15—C16	105.4 (2)
C2—O1—H1	101.8 (19)	O5—C16—C17	115.6 (3)
C3—O2—H2	113 (4)	C15—C16—C17	135.0 (3)
C7—N1—C8	122.0 (2)	O5—C16—C15	109.4 (3)
S1—N2—C14	123.00 (17)	C3—C4—H4	119.00
O5—N3—C14	104.68 (19)	C5—C4—H4	119.00
C14—N2—H2A	114 (2)	C4—C5—H5	120.00
S1—N2—H2A	113.1 (17)	C6—C5—H5	120.00
C6—C1—C7	120.8 (3)	C1—C6—H6	120.00
C2—C1—C7	120.2 (3)	C5—C6—H6	120.00
C2—C1—C6	119.0 (3)	N1—C7—H7	119.00
O1—C2—C3	117.5 (3)	C1—C7—H7	119.00
C1—C2—C3	119.8 (3)	C8—C9—H9	119.00
O1—C2—C1	122.7 (3)	C10—C9—H9	120.00
O2—C3—C2	120.6 (3)	C9—C10—H10	120.00
O2—C3—C4	119.7 (3)	C11—C10—H10	120.00
C2—C3—C4	119.7 (3)	C11—C12—H12	120.00
C3—C4—C5	121.1 (3)	C13—C12—H12	120.00
C4—C5—C6	119.6 (3)	C8—C13—H13	120.00
C1—C6—C5	120.7 (3)	C12—C13—H13	120.00
N1—C7—C1	122.7 (3)	C14—C15—H15	127.00
N1—C8—C9	117.0 (3)	C16—C15—H15	127.00
N1—C8—C13	124.0 (3)	C16—C17—H17A	109.00
C9—C8—C13	119.0 (3)	C16—C17—H17B	109.00
C8—C9—C10	121.0 (3)	C16—C17—H17C	109.00
C9—C10—C11	119.4 (3)	H17A—C17—H17B	109.00
S1—C11—C12	120.56 (19)	H17A—C17—H17C	109.00
C10—C11—C12	120.0 (2)	H17B—C17—H17C	110.00
			
O3—S1—N2—C14	−48.5 (2)	C2—C1—C7—N1	−1.1 (4)
O4—S1—N2—C14	−177.4 (2)	C6—C1—C7—N1	179.6 (3)
C11—S1—N2—C14	67.9 (2)	O1—C2—C3—O2	−0.8 (4)
O3—S1—C11—C10	−174.45 (19)	O1—C2—C3—C4	−179.9 (3)
O3—S1—C11—C12	5.1 (2)	C1—C2—C3—O2	179.9 (3)
O4—S1—C11—C10	−41.7 (2)	C1—C2—C3—C4	0.8 (5)
O4—S1—C11—C12	137.8 (2)	O2—C3—C4—C5	−179.7 (3)
N2—S1—C11—C10	69.8 (2)	C2—C3—C4—C5	−0.6 (5)
N2—S1—C11—C12	−110.7 (2)	C3—C4—C5—C6	−0.2 (5)
C16—O5—N3—C14	−0.8 (3)	C4—C5—C6—C1	0.9 (5)
N3—O5—C16—C15	0.0 (3)	N1—C8—C9—C10	179.8 (2)
N3—O5—C16—C17	−178.6 (2)	C13—C8—C9—C10	−1.6 (4)
C8—N1—C7—C1	−179.0 (2)	N1—C8—C13—C12	−179.5 (3)
C7—N1—C8—C9	−163.9 (3)	C9—C8—C13—C12	2.0 (4)
C7—N1—C8—C13	17.6 (4)	C8—C9—C10—C11	0.8 (4)
S1—N2—C14—N3	−146.5 (2)	C9—C10—C11—S1	179.3 (2)
S1—N2—C14—C15	36.6 (4)	C9—C10—C11—C12	−0.3 (4)
O5—N3—C14—N2	−176.12 (19)	S1—C11—C12—C13	−178.9 (2)
O5—N3—C14—C15	1.3 (3)	C10—C11—C12—C13	0.7 (4)
C6—C1—C2—O1	−179.4 (2)	C11—C12—C13—C8	−1.6 (4)
C6—C1—C2—C3	−0.2 (4)	N2—C14—C15—C16	175.6 (2)
C7—C1—C2—O1	1.2 (4)	N3—C14—C15—C16	−1.3 (3)
C7—C1—C2—C3	−179.6 (3)	C14—C15—C16—O5	0.8 (3)
C2—C1—C6—C5	−0.7 (4)	C14—C15—C16—C17	179.0 (3)
C7—C1—C6—C5	178.7 (3)		

### Hydrogen-bond geometry (Å, º)

**Table:**

*D*—H···*A*	*D*—H	H···*A*	*D*···*A*	*D*—H···*A*
O1—H1···N1	0.99 (4)	1.66 (4)	2.588 (3)	155 (3)
O2—H2···O4^i^	0.79 (4)	2.11 (5)	2.848 (3)	154 (5)
N2—H2*A*···N3^ii^	0.84 (3)	2.05 (3)	2.881 (3)	172 (3)

Symmetry codes: (i) −*x*, −*y*+1, −*z*+1; (ii) −*x*+1, −*y*+1, −*z*+2.
